# 2-(4-Chloro­anilino)-1-phenyl­ethanone

**DOI:** 10.1107/S1600536811006404

**Published:** 2011-02-26

**Authors:** Xing-Jun Yao, Qian Yuan

**Affiliations:** a College of Chemistry and Chemical Engineering, Liaocheng University, 252059 Liaocheng, Shandong, People’s Republic of China; b Guodian Liaocheng Power Co. Ltd, 252033 Liaocheng, Shandong, People’s Republic of China

## Abstract

In the title compound, C_14_H_12_ClNO, the planes of the two aromatic rings form a dihedral angle of 4.16 (1)°. The mol­ecule is essentially planar with an r.m.s. deviation for all non-H atoms of 0.0372 Å.

## Related literature

For a related structure, see: Anilkumar *et al.* (2005 ▶).
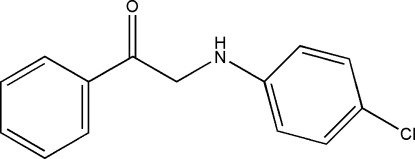


## Experimental

### 

#### Crystal data

C_14_H_12_ClNO
*M* *_r_* = 245.70
Triclinic, 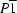

*a* = 5.6500 (5) Å
*b* = 7.3921 (8) Å
*c* = 13.9769 (14) Å
α = 98.588 (2)°
β = 91.095 (1)°
γ = 97.590 (1)°
*V* = 571.70 (10) Å^3^
*Z* = 2
Mo *K*α radiation
μ = 0.31 mm^−1^
*T* = 298 K
0.15 × 0.12 × 0.10 mm

#### Data collection

Bruker SMART APEX diffractometer
Absorption correction: multi-scan (*SADABS*; Bruker, 2005 ▶) *T*_min_ = 0.954, *T*_max_ = 0.969
2924 measured reflections
1971 independent reflections
1238 reflections with *I* > 2σ(*I*)
*R*_int_ = 0.032

#### Refinement

*R*[*F* ^2^ > 2σ(*F* ^2^)] = 0.061
*wR*(*F* ^2^) = 0.178
*S* = 1.03
1971 reflections
154 parameters
H-atom parameters constrained
Δρ_max_ = 0.44 e Å^−3^
Δρ_min_ = −0.36 e Å^−3^

### 

Data collection: *APEX2* (Bruker, 2005 ▶); cell refinement: *SAINT* (Bruker, 2005 ▶); data reduction: *SAINT*; program(s) used to solve structure: *SHELXTL* (Sheldrick, 2008 ▶); program(s) used to refine structure: *SHELXTL*; molecular graphics: *SHELXTL*; software used to prepare material for publication: *SHELXTL*.

## Supplementary Material

Crystal structure: contains datablocks global, I. DOI: 10.1107/S1600536811006404/ng5121sup1.cif

Structure factors: contains datablocks I. DOI: 10.1107/S1600536811006404/ng5121Isup2.hkl

Additional supplementary materials:  crystallographic information; 3D view; checkCIF report

## Figures and Tables

**Fig. 1.**
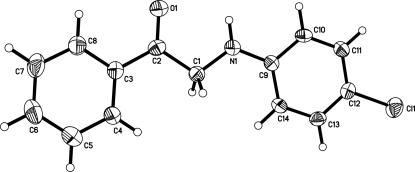
View of the title compound showing the atomic labeling and 30% probability displacement ellipsoids.

## supplementary crystallographic information

### Comment

The title compound, C_14_H_12_ClNO, is often used as catalyst for the polymerization of olefins and as a reactant in organic synthesis. The molecule of (I) is shown in Fig. 1. The bond lengths and angles are normal. The dihedral angle between the two benzene rings is 4.16 (1) °. The molecule is essentially planar, the r.m.s. deviation for all non-H atoms being 0.0372 Å.

### Experimental

The title compound was synthesized by the reaction of 4-chloroaniline (1 mmol, 127.6 mg) with 2-bromo-1-phenylethanone (1 mmol, 199.0 mg) in ethanol (20 ml) under reflux conditions (338 K) for 3 h. The solvent was removed and the solid product recrystallized from ethanol. After six days brown crystals were obtained that were suitable for X-ray diffraction study.

### Refinement

All H atoms were placed in idealized positions (C—H = 0.93–0.97 Å, N—H = 0.86 Å) and refined as riding atoms. For those bound to C, *U*_iso_(H) = 1.2 or 1.5 *U*_eq_(C) whereas for those bound to N, *U*_iso_(H) = 1.5 *U*_eq_(N).

### Crystal data

**Table:**

C_14_H_12_ClNO	*Z* = 2
*M**_r_* = 245.70	*F*(000) = 256
Triclinic, *P*1	*D*_x_ = 1.427 Mg m^−^^3^
Hall symbol: -P 1	Mo *K*α radiation, λ = 0.71073 Å
*a* = 5.6500 (5) Å	Cell parameters from 945 reflections
*b* = 7.3921 (8) Å	θ = 2.8–24.4°
*c* = 13.9769 (14) Å	µ = 0.31 mm^−^^1^
α = 98.588 (2)°	*T* = 298 K
β = 91.095 (1)°	Block, brown
γ = 97.590 (1)°	0.15 × 0.12 × 0.10 mm
*V* = 571.70 (10) Å^3^	

### Data collection

**Table:**

Bruker SMART APEX diffractometer	1971 independent reflections
Radiation source: fine-focus sealed tube	1238 reflections with *I* > 2σ(*I*)
graphite	*R*_int_ = 0.032
φ and ω scans	θ_max_ = 25.1°, θ_min_ = 2.8°
Absorption correction: multi-scan (*SADABS*; Bruker, 2005)	*h* = −6→6
*T*_min_ = 0.954, *T*_max_ = 0.969	*k* = −7→8
2924 measured reflections	*l* = −13→16

### Refinement

**Table:**

Refinement on *F*^2^	Primary atom site location: structure-invariant direct methods
Least-squares matrix: full	Secondary atom site location: difference Fourier map
*R*[*F*^2^ > 2σ(*F*^2^)] = 0.061	Hydrogen site location: inferred from neighbouring sites
*wR*(*F*^2^) = 0.178	H-atom parameters constrained
*S* = 1.03	*w* = 1/[σ^2^(*F*_o_^2^) + (0.1*P*)^2^ + 0.0005*P*] where *P* = (*F*_o_^2^ + 2*F*_c_^2^)/3
1971 reflections	(Δ/σ)_max_ < 0.001
154 parameters	Δρ_max_ = 0.44 e Å^−^^3^
0 restraints	Δρ_min_ = −0.36 e Å^−^^3^

### Special details

**Table:**

Geometry. All e.s.d.'s (except the e.s.d. in the dihedral angle between two l.s. planes)are estimated using the full covariance matrix. The cell e.s.d.'s are takeninto account individually in the estimation of e.s.d.'s in distances, anglesand torsion angles; correlations between e.s.d.'s in cell parameters are onlyused when they are defined by crystal symmetry. An approximate (isotropic)treatment of cell e.s.d.'s is used for estimating e.s.d.'s involving l.s. planes.
Refinement. Refinement of *F*^2^ against ALL reflections. The weighted *R*-factor *wR* and goodness of fit *S* are based on *F*^2^, conventional *R*-factors *R* are based on *F*, with *F* set to zero for negative *F*^2^. The threshold expression of *F*^2^ > σ(*F*^2^) is used only for calculating *R*-factors(gt) *etc*. and is not relevant to the choice of reflections for refinement. *R*-factors based on *F*^2^ are statistically about twice as large as those based on *F*, and *R*- factors based on ALL data will be even larger.

### Fractional atomic coordinates and isotropic or equivalent isotropic displacement parameters (Å^2^)

**Table:**

	*x*	*y*	*z*	*U*_iso_*/*U*_eq_	
Cl1	−0.43122 (17)	0.65041 (13)	0.83483 (7)	0.0650 (4)	
N1	0.1395 (5)	0.7998 (4)	0.5053 (2)	0.0506 (8)	
H1	0.2852	0.8498	0.5179	0.061*	
O1	0.4464 (4)	0.8715 (3)	0.37523 (18)	0.0598 (7)	
C1	0.0566 (6)	0.7594 (4)	0.4079 (2)	0.0413 (8)	
H1A	−0.0764	0.8266	0.3988	0.050*	
H1B	−0.0005	0.6285	0.3920	0.050*	
C2	0.2487 (6)	0.8107 (4)	0.3420 (2)	0.0415 (8)	
C3	0.1916 (5)	0.7858 (4)	0.2383 (2)	0.0405 (8)	
C4	−0.0282 (6)	0.7083 (4)	0.1998 (2)	0.0533 (9)	
H4	−0.1467	0.6676	0.2399	0.064*	
C5	−0.0752 (7)	0.6900 (5)	0.1028 (3)	0.0706 (12)	
H5	−0.2255	0.6365	0.0769	0.085*	
C6	0.0965 (8)	0.7495 (5)	0.0435 (3)	0.0737 (12)	
H6	0.0634	0.7377	−0.0228	0.088*	
C7	0.3167 (8)	0.8265 (5)	0.0813 (3)	0.0700 (12)	
H7	0.4348	0.8671	0.0410	0.084*	
C8	0.3635 (6)	0.8438 (4)	0.1780 (2)	0.0536 (10)	
H8	0.5146	0.8959	0.2036	0.064*	
C9	0.0012 (5)	0.7637 (4)	0.5807 (2)	0.0363 (7)	
C10	0.0950 (6)	0.8121 (4)	0.6740 (2)	0.0424 (8)	
H10	0.2520	0.8695	0.6842	0.051*	
C11	−0.0353 (6)	0.7783 (4)	0.7514 (2)	0.0446 (8)	
H11	0.0313	0.8129	0.8139	0.054*	
C12	−0.2643 (6)	0.6932 (4)	0.7368 (2)	0.0412 (8)	
C13	−0.3618 (6)	0.6439 (4)	0.6462 (3)	0.0449 (8)	
H13	−0.5185	0.5854	0.6369	0.054*	
C14	−0.2313 (6)	0.6795 (4)	0.5679 (2)	0.0445 (8)	
H14	−0.3004	0.6466	0.5057	0.053*	

### Atomic displacement parameters (Å^2^)

**Table:**

	*U*^11^	*U*^22^	*U*^33^	*U*^12^	*U*^13^	*U*^23^
Cl1	0.0711 (7)	0.0651 (7)	0.0599 (7)	0.0021 (5)	0.0184 (5)	0.0176 (5)
N1	0.0441 (17)	0.0607 (19)	0.0426 (18)	−0.0048 (14)	0.0015 (13)	0.0045 (14)
O1	0.0500 (16)	0.0705 (18)	0.0540 (16)	−0.0095 (13)	−0.0005 (13)	0.0097 (13)
C1	0.0433 (19)	0.0377 (19)	0.042 (2)	0.0048 (15)	0.0009 (16)	0.0047 (15)
C2	0.044 (2)	0.0269 (17)	0.053 (2)	0.0003 (15)	0.0030 (17)	0.0074 (14)
C3	0.050 (2)	0.0285 (17)	0.043 (2)	0.0070 (15)	0.0045 (16)	0.0044 (14)
C4	0.054 (2)	0.060 (2)	0.044 (2)	0.0007 (18)	0.0003 (18)	0.0076 (17)
C5	0.069 (3)	0.082 (3)	0.057 (3)	0.003 (2)	−0.011 (2)	0.006 (2)
C6	0.099 (4)	0.077 (3)	0.045 (2)	0.009 (3)	−0.001 (2)	0.015 (2)
C7	0.091 (3)	0.062 (3)	0.057 (3)	−0.003 (2)	0.020 (2)	0.018 (2)
C8	0.063 (2)	0.044 (2)	0.051 (2)	−0.0021 (17)	0.0072 (18)	0.0044 (16)
C9	0.0419 (18)	0.0278 (17)	0.0393 (19)	0.0074 (14)	0.0003 (15)	0.0032 (13)
C10	0.0364 (17)	0.0358 (19)	0.053 (2)	−0.0030 (14)	−0.0031 (16)	0.0059 (15)
C11	0.050 (2)	0.044 (2)	0.0389 (19)	0.0025 (16)	−0.0033 (16)	0.0058 (15)
C12	0.046 (2)	0.0348 (18)	0.045 (2)	0.0078 (15)	0.0086 (16)	0.0104 (14)
C13	0.0369 (18)	0.041 (2)	0.054 (2)	−0.0004 (15)	0.0013 (16)	0.0021 (16)
C14	0.043 (2)	0.048 (2)	0.0388 (19)	0.0025 (16)	−0.0054 (16)	−0.0010 (15)

### Geometric parameters (Å, °)

**Table:**

Cl1—C12	1.723 (3)	C6—C7	1.360 (5)
N1—C9	1.361 (4)	C6—H6	0.9300
N1—C1	1.407 (4)	C7—C8	1.356 (5)
N1—H1	0.8600	C7—H7	0.9300
O1—C2	1.204 (4)	C8—H8	0.9300
C1—C2	1.485 (4)	C9—C14	1.374 (4)
C1—H1A	0.9700	C9—C10	1.376 (5)
C1—H1B	0.9700	C10—C11	1.355 (4)
C2—C3	1.457 (4)	C10—H10	0.9300
C3—C4	1.362 (4)	C11—C12	1.359 (5)
C3—C8	1.367 (4)	C11—H11	0.9300
C4—C5	1.360 (4)	C12—C13	1.350 (5)
C4—H4	0.9300	C13—C14	1.370 (4)
C5—C6	1.359 (4)	C13—H13	0.9300
C5—H5	0.9300	C14—H14	0.9300
			
C9—N1—C1	123.4 (3)	C8—C7—C6	119.8 (3)
C9—N1—H1	118.3	C8—C7—H7	120.1
C1—N1—H1	118.3	C6—C7—H7	120.1
N1—C1—C2	111.2 (3)	C7—C8—C3	120.9 (3)
N1—C1—H1A	109.4	C7—C8—H8	119.6
C2—C1—H1A	109.4	C3—C8—H8	119.6
N1—C1—H1B	109.4	N1—C9—C14	122.6 (3)
C2—C1—H1B	109.4	N1—C9—C10	119.6 (3)
H1A—C1—H1B	108.0	C14—C9—C10	117.8 (3)
O1—C2—C3	121.9 (3)	C11—C10—C9	121.8 (3)
O1—C2—C1	119.4 (3)	C11—C10—H10	119.1
C3—C2—C1	118.7 (3)	C9—C10—H10	119.1
C4—C3—C8	119.0 (3)	C10—C11—C12	119.3 (3)
C4—C3—C2	122.2 (3)	C10—C11—H11	120.3
C8—C3—C2	118.9 (3)	C12—C11—H11	120.3
C5—C4—C3	120.3 (3)	C13—C12—C11	120.4 (3)
C5—C4—H4	119.9	C13—C12—Cl1	120.0 (3)
C3—C4—H4	119.9	C11—C12—Cl1	119.6 (3)
C6—C5—C4	120.3 (4)	C12—C13—C14	120.4 (3)
C6—C5—H5	119.9	C12—C13—H13	119.8
C4—C5—H5	119.9	C14—C13—H13	119.8
C5—C6—C7	119.9 (4)	C13—C14—C9	120.3 (3)
C5—C6—H6	120.1	C13—C14—H14	119.9
C7—C6—H6	120.1	C9—C14—H14	119.9
			
C9—N1—C1—C2	−178.9 (3)	C2—C3—C8—C7	−178.8 (3)
N1—C1—C2—O1	2.6 (4)	C1—N1—C9—C14	1.8 (5)
N1—C1—C2—C3	−177.4 (2)	C1—N1—C9—C10	−178.3 (3)
O1—C2—C3—C4	176.5 (3)	N1—C9—C10—C11	−179.7 (3)
C1—C2—C3—C4	−3.5 (4)	C14—C9—C10—C11	0.1 (5)
O1—C2—C3—C8	−4.2 (4)	C9—C10—C11—C12	0.5 (5)
C1—C2—C3—C8	175.8 (2)	C10—C11—C12—C13	−0.4 (5)
C8—C3—C4—C5	−0.3 (2)	C10—C11—C12—Cl1	−179.8 (2)
C2—C3—C4—C5	179.0 (3)	C11—C12—C13—C14	−0.2 (5)
C3—C4—C5—C6	−0.2 (2)	Cl1—C12—C13—C14	179.2 (2)
C4—C5—C6—C7	0.5 (4)	C12—C13—C14—C9	0.8 (5)
C5—C6—C7—C8	−0.2 (5)	N1—C9—C14—C13	179.0 (3)
C6—C7—C8—C3	−0.4 (5)	C10—C9—C14—C13	−0.8 (5)
C4—C3—C8—C7	0.6 (4)		
